# Mitigating Mismatch Compression in Differential Local Field Potentials

**DOI:** 10.1109/TNSRE.2022.3217469

**Published:** 2023-01-30

**Authors:** Vineet Tiruvadi, Samuel James, Bryan Howell, Mosadoluwa Obatusin, Andrea Crowell, Patricio Riva-Posse, Robert E. Gross, Cameron C. McIntyre, Helen S. Mayberg, Robert Butera

**Affiliations:** Department of Biomedical Engineering, School of Electrical and Computer Engineering, Georgia Institute of Technology, Atlanta, GA 30332 USA, and also with the Emory School of Medicine, Atlanta, GA 30307 USA; School of Electrical and Computer Engineering, Georgia Institute of Technology, Atlanta, GA 30332 USA.; Department of Biomedical Engineering, Duke University, Durham, NC 27708 USA.; Icahn School of Medicine at Mount Sinai, New York, NY 10029 USA.; Emory School of Medicine, Atlanta, GA 30307 USA.; Emory School of Medicine, Atlanta, GA 30307 USA.; Department of Biomedical Engineering, Georgia Institute of Technology, Atlanta, GA 30332 USA, and also with the Emory School of Medicine, Atlanta, GA 30307 USA.; Department of Biomedical Engineering, Duke University, Durham, NC 27708 USA.; Icahn School of Medicine at Mount Sinai, New York, NY 10029 USA.; Department of Biomedical Engineering, Georgia Institute of Technology, Atlanta, GA 30332 USA.

**Keywords:** Deep brain stimulation, adaptive, readout, mismatch compression, clinical electrophysiology

## Abstract

Deep brain stimulation (DBS) devices capable of measuring differential local field potentials (∂LFP) enable neural recordings alongside clinical therapy. Efforts to identify oscillatory correlates of various brain disorders, or *disease readouts*, are growing but must proceed carefully to ensure readouts are not distorted by brain environment. In this report we identified, characterized, and mitigated a major source of distortion in ∂LFP that we introduce as *mismatch compression* (MC). Using *in vivo, in silico*, and *in vitro* models of MC, we showed that impedance mismatches in the two recording electrodes can yield incomplete rejection of stimulation artifact and subsequent gain compression that distorts oscillatory power. We then developed and validated an opensource mitigation pipeline that mitigates the distortions arising from MC. This work enables more reliable oscillatory readouts for adaptive DBS applications.

## I. Introduction

DEEP brain stimulation (DBS) has demonstrated efficacy in various neuropsychiatric disorders [1], [2], [3] but our understanding of its effects on brain signaling remains lacking. New devices that measure neural recordings directly in DBS patients are being used around [4], and even during [5], [6], [7], [8], [9], [10], active DBS therapy in order to identify correlates of diseases, or *disease readouts*. However, these new devices require caution to ensure any derived disease readout is reliable and stable over long periods of time.

**Fig. 1.**
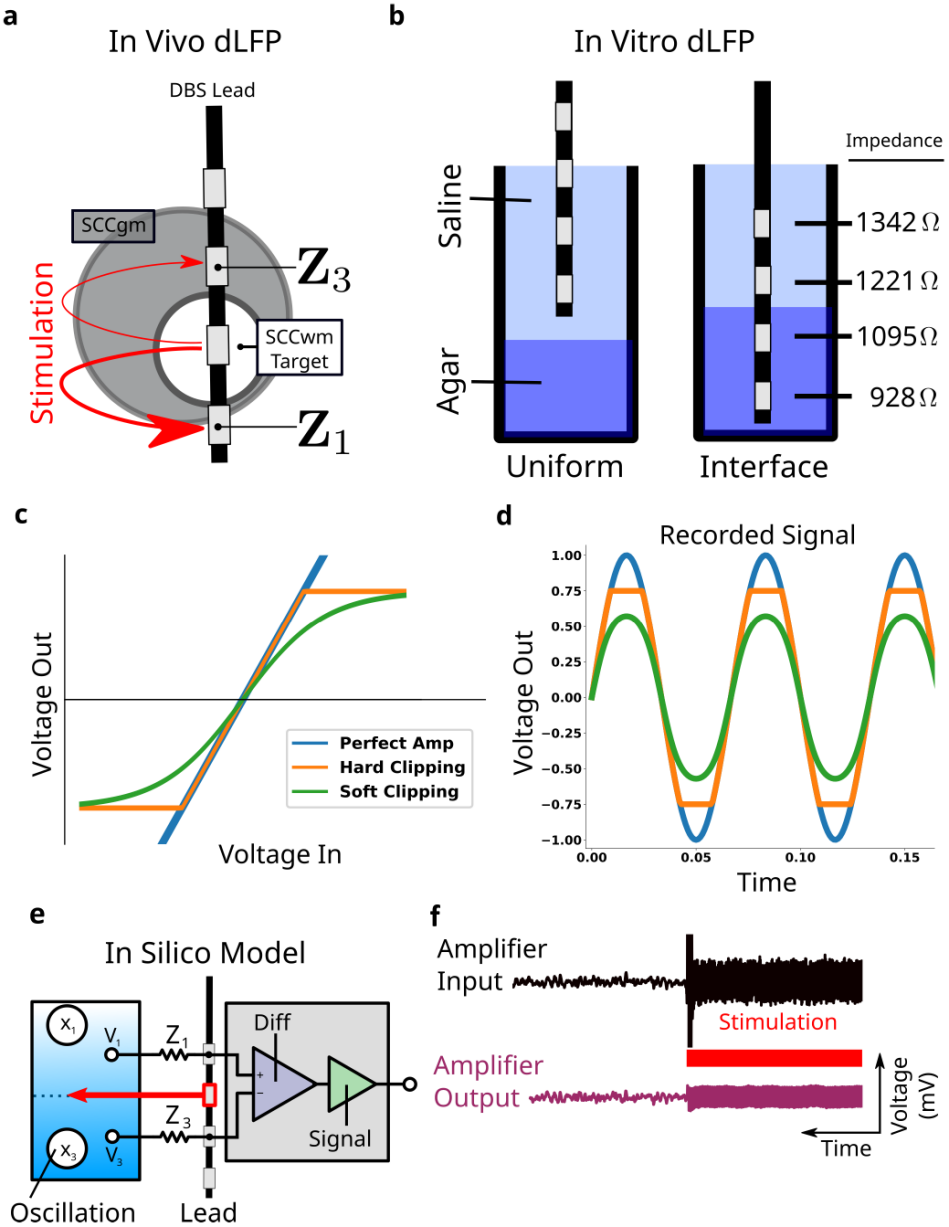
Mismatch Compression in ∂LFPs. **a**, Clinical DBS leads have four electrodes, each of which can be in either gray or white matter. ∂LFP records from two electrodes around the stimulation electrode. **b**, An *in vitro* model introduces differences in resistivity to test whether impedance mismatches in the two electrodes causes gain compression in amplifiers. **c**, Amplifier transfer function shows how inputs are transformed into outputs. Three models are simulated here. **d**, Time domain output from the different amplifier models demonstrate the effects of gain compression, both hard-clipping and soft-clipping. **e**, Schematic of the focused MC model consists of brain, lead, amplifier layers. **f**, Simulated ∂LFP is analysed with the same approaches used for empirical measurements.

A new class of DBS capable of *differential* local field potential (∂LFP) recordings enables long-term recording around the stimulation target [6], [7]. Unlike typical LFP recordings, ∂LFP references two electrodes around the stimulation electrode to each other in order to subtract away large artifacts before sensitive amplifiers (Figure 1a) [7], [11]. Without subtraction, artifacts can leak into sensitive amplifiers and cause saturation, an extreme form of a more general process called *gain compression* (Figure 1c,d) [12]. Because gain compression may be overt or subtle (Figure 1c,d), analyses in the frequency-domain to identify the high-frequency changes associated with compression is needed to confirm and characterize it when present.

While ∂LFP can be very effective, even small asymmetries in recording electrodes can result in leaked artifacts many orders of magnitude larger than neural oscillations [7], [13]. These asymmetries in electrical *impedances* or surrounding tissue types, can differentially affect stimulation spread and field potential recordings [14], [15], [16], [17], [18]. Changes in impedance over weeks and months of symptom recovery can, through variability in resulting gain compression, confound analyses of neural oscillations [9], [15], [16], [19], [20] - a process we study here as *mismatch compression* (MC).

In this study we characterize MC in a first-generation ∂LFP recording device, the Activa PC+S^™^ (Medtronic PLC, Minnesota, USA) [7]. Our core goal here was to mitigate the effects of MC for more reliable analyses of oscillations in ∂LFP recordings, particularly in the presence of active stimulation and over chronic timescales. We collected a set of clinical recordings from patients with treatment resistant depression (TRD) implanted with connectomics-guided DBS at subcallosal cingulate white matter (SCCwm), a brain target with known tissue heterogeneity in the volume of tissue activated (VTA) [2], [21], [22], [23]. Using multiple model systems, we developed an equivalent circuit, or focused model, of MC to confirm its presence, characterized its distortions to frequency-domain features, and validated a mitigation pipeline that avoid MC distortions for more reliable oscillatory analyses.

## II. Methods

### A. Clinical Protocol

Six patients with treatment resistant depression (TRD) were consented and enrolled in an IRB and FDA approved research protocol at Emory University (clinicaltrials.gov NCT01984710; FDA IDE G130107) (Table I). All six patients were implanted with a Medtronic Activa PC+S^™^ implantable pulse generator (IPG), a first-generation ∂LFP device used for opportunistic study of brain activity in clinical DBS [6]. Two Medtronic DBS3387 leads, each with four electrodes, were implanted bilaterally in patient-specific, tractographydefined SCCwm as previously described (Figure 1a) [24]. Patients underwent weekly clinical assessment, including DBS electrode impedance measurements and downloads of the previous week’s daily recordings.

### B. Stimulation and Impedance

In each hemisphere’s SCC, stimulation was delivered to one of four electrodes spaced 1.5 mm apart edge-to-edge Therapeutic DBS stimulation is delivered bilaterally at 130 Hz, 90 μs pulsewidth, biphasic waveform with IPG as cathode. Stimulation voltage is varied depending on clinical efficacy and experimental condition. All electrode impedances were measured in monopolar mode at 3 V and 100 Hz using the standard clinician-controller (Medtronic N’Vision) [25]. Impedance measurements were made at weekly clinical patient assessments over 28 weeks post-implantation. Impedance mismatch was calculated as the absolute value of the subtracted impedances in both electrodes each week.

### C. Clinical (in Vivo) Recordings

#### 1) Channel Sampling and Filters:

Each DBS lead has four electrodes, with those on the left lead labeled E0-3 and the right lead labeled E8-E11 (Figure 1a). In this study, all recordings were taken with the electrodes directly adjacent to the therapeutic electrodes (Figure 1a). All PC+S^™^recordings were sampled at the dual-channel maximum of 422 Hz. Hardware filters with limited settings were set as wide as possible, with 0.5 Hz high-pass and 100 Hz low-pass. ***In vivo***, recordings were taken from bilateral SCC using patient-specific parameters for recording electrode number and channel gains (Table I). ***In vitro***, a single DBS 3387 lead was connected to the channels 0-7 on the Activa PC+S^™^. All recordings were collected using the clinical sensing tablet using clinical recording parameters settings.

#### 2) Gain and Over Range Marker (ORM):

Each channel has an adjustable gain parameter, selected from 250, 500, 1000, or 2000, set after visual inspection of the recorded spectrum during therapeutic stimulation (Table I). A PC+S^™^ specific *over range marker*(ORM) is present in all recordings at 105.5 Hz. The ORM, a constant amplitude signal in the recording, can be seen in the acute voltage sweep experiment across the full recording, with power changes evident during active stimulation (Figure 4a). This observed variability is indicative of gain compression, with saturation considered when the peak is no longer discernible (Figure 4a at 800 s).

#### 3) Voltage Sweep Experiment:

A voltage sweep experiment was performed in all patients at therapy initiation. Stimulation is delivered to the therapeutic electrode at voltages 2V to 8V, with otherwise therapeutic stimulation parameters (See Methods II-C.3). Each stimulation condition was delivered for 1 min, with 1 min washout periods in between.

### D. Benchtop (in Vitro) Construct

An agar-saline preparation of two spatially sequestered phases with distinct resistivity (Figure 1b) was constructed based on published experimental setups [26]. The saline phase was fixed at $0.5\frac{\text{mg}}{\text{mL}}$ of NaCl, and yielded measured impedances of approximately 800 Ω. The agar phase was fixed with high resistivity $0.1\frac{\text{mg}}{\text{mL}}$ of NaCl), and yielded measured impedances of approximately 1300 Ω. Agar mixture was poured into a 10 mL conical corning tube with blue fluorophore and placed in a 32C for 20 minutes to settle before saline phase was then added on top (Figure 4a). A demo DBS3387 lead was placed at the interface of the saline and agar layers using a micromanipulator (Figure 4b). Impedance mismatches measured across two non-adjacent electrodes ranged from 100 Ω in uniform media and 300 Ω across media (Figure 1b).

### E. Focused (in Silico) Model

Gain compression was a central design challenge in the development of the PC+S^™^, and impedance mismatches in the recordings electrodes was an expected failure mode by device engineers [7]. Because of the deliberate design of ∂LFP channels, we can construct an explicit, computational model of MC in order to confirm its presence, characterize its distortions, and mitigate its distortions as best as possible. Our focused MC model consisted of four layers: the brain, the lead, the amplifier, and the analysis (Figure 1d).

#### 1) Brain Layer:

All brain signals are generated at 4220 Hz timeseries lasting 20 s. A single neural oscillation *x*_3_ was implemented as a stationary 15 Hz sinusoid (Figure 1d), and an independent $\frac{1}{f}$ components added to *x*_1_ and *x*_3_ sources. Stimulation artifact was introduced as a truncated Fourier series of sine waves at the therapeutic stimulation *f*_*T*_, yielding the *stimulation shaping harmonics* (SSH). The stimulation *S(t)* is set six orders of magnitude larger than the neural oscillations, based on the typical difference between neural oscillations *μ*V and stimulation V [13], [27]. The Activa PC+S^™^ specific ORM is added at 105.5 Hz in order to improve congruence between simulation and empirical recordings.

#### 2) Lead Layer:

The lead layer consists of two electrodes, electrodes *e*_1_ and *e*_3_, around the stimulating electrode *e*_2_. Both measure independently from two neural sources, labeled *x*_1_ and *x*_3_ for electrodes *e*_1_ and *e*_3_, respectively. The neural sources are independent of each other, with only *x*_1_ containing an oscillation at 15 Hz. Each electrode has a modeled impedance as a pure resistive component *Z*_1_ and *Z*_3_, respectively.

#### 3) Amplifier Layer:

Two subcomponents are implemented in the amplifier layer: a differential amplifier and a signal amplifier. The differential amplifier gains the difference of the two inputs, with the common-mode rejection ratio modeled as ∞ [7]. The output of the ideal differential amplifier then goes through a signal amplifier, modeled as either a perfect amp (fully linear), hard-clipping (piece-wise linear), or soft-clipping (tanh function), corresponding to the different models of saturation used clinically (Figure 1c). Soft-clipping yields the most realistic gain compression and can yield soft-clipping not discernible by eye (Figure 1d).


(1) $$V_{\text{lfp}}=g_{2}\cdot \tanh(g_{1}\cdot V_{\text{out}})$$

Final subsampling by the analog-digital-converter (ADC) is modeled as a 10-fold downsample yielding a simulated *partial*LFP at 422 Hz, matching the settings of the PC+S^™^.

#### 4) Analysis Layer:

The final simulated ∂LFP (Figure 1e) was analysed in the frequency-domain for oscillatory power changes with the same approach taken for empirical recordings (See Methods II-G). Gain compression is associated mainly with two frequency-domain distortions: broad-spectrum slope flattening and narrowband oscillatory artifacts [12], [28], [29]. Broad-spectrum slope flattening is visually distinct and fit on the logPSD using a polynomial of fourth order, shown to be informative in isolating oscillations [30], [31]. The narrowband distortions will be classified into three categories. First, the *stimulation shaping harmonics* that is the higher-frequency content that shapes the stimulation waveform beyond its 130 Hz base frequency. Second, the *aliased shaping harmonics* (ASH) that are the alises of the SSH above Nyquist rate (211 Hz). Third, the *intermodulation harmonics* (IMH) that arise from compressing the stimulation waveform.

### F. Model Parameters

Model parameters (Table II) were chosen to yield simulated ∂LFP with MC-salient phenomenological features, particularly broad spectrum shape. *A_d_* is amplitude of the stimulation artifact after common-mode rejection, which is set at 0.5 V to reflect a 10% stimulation leakage at typical impedance mismatches. *Z_b_* is the internal impedance of the differential channel, set based on communications with PC+S^™^ engineers to reflect an approximate equivalent impedance. *x*_1_ amplitude is the strength of the oscillation, set at 2 *μ*V to reflect strong neural oscillation. *g*1 is the post-differential amplifier gain, *g*2 is the post-signal amplifier gain, both normalized to 1 for simplicity.

### G. Oscillatory Analyses

All analyses were performed with a custom Python library, named dbspace, available through PyPI. ∂LFP were transformed to the frequency domain using a Welch power spectral density (PSD) estimate with 1024 FFT bins, 0% overlap, 844 sample Blackman-Harris Window. PSDs were log-transformed 10 · log_10_(*P*_*xx*_) to visualize logPSD and perform preprocessing. Oscillatory power was then computed as either the mean or median value of the PSD for a predefined frequency range corresponding to standard oscillatory bands: *δ* (1 Hz to 4 Hz), *θ* (4 Hz to 8 Hz),, *α* (8 Hz to 14 Hz), *β* (14 Hz to 30 Hz), *γ* (30 Hz to 50 Hz) [4].

### H. Mitigation Pipeline for MC

MC mitigation consists of a pipeline with four main steps performed on the log-transformed power spectral density (PSD; Figure 3). First, a fourth-order polynomial is fit and subtraction to remove broad-spectrum changes (Figure 3b, dotted orange line). Second, the frequency bands of oscillations are used to calculate power, with specific frequencies chosen to avoid distortions predicted in the focused MC model without extending a band’s traditional frequency range (Table III). Third, the median power across frequency bins is taken instead of the mean to mitigate remaining narrowband artifacts.

### I. Gain Compression Ratio

A *gain compression ratio* (GCr) can be calculated on the original recording to determine the level of gain compression occurring. Comparing the power in the ASH artifacts (64 Hz) versus the IMH (66 Hz) (Figure 6h) measures the level of gain compression. Gain compression ratio (GCr) is calculated as the log-ratio of power in the ASH (62 Hz)vs IMH (64 Hz).

### J. Analysis and Simulation Code

The MC model is developed in a custom Python library and run across a set of scripts that integrate simulation with empirical analyses. Analyses and simulation were done through open-source Jupyter Notebooks available at https://github.com/virati/mismatch_compression. Dependencies and associated libraries are available through PyPi: NumPy [32], SciPy [33], Allantools [34], and dbspace [35].

## III. Results

### A. Clinical ∂LFP Demonstrate Significant Variability

First, we inspect the clinical *in vivo* recordings to identify visible frequency-domain changes. Significant variability in the PSD was found in both voltage sweep experiments (Figure 4a) and weekly averaged recordings (Figure 4b). The primary changes observed were broad-spectrum increases in power and the emergence of numerous narrow-band peaks. In the voltage sweep, these changes are time-locked to active DBS and increase with the DBS amplitude (Figure 4a). In the chronic recordings, significant broad-spectrum variability is found across 24 weeks of weekly average recordings (Figure 4b).

### B. DBS Electrodes Have Dynamic Properties

Weekly impedance mismatches ranged between 0 Ω and 600 Ω with significant variability across time, between patients, and between leads (Figure 4c,d). Left and right impedances changed differently from each other, with left having more variability across patients than right. Two distinct dynamics are observed in the impedance mismatch: large week-to-week changes in the first 10 weeks, and stable beyond 10 weeks. The measured power in constant amplitude ORM can reflect saturation, and we observed large variability in the ORM power across time in each patient, across patients, and across hemispheres (Figure 4e,f). The ORM demonstrates large changes throughout the study, with the chronic therapy onset at 4 weeks consistently associated, across patient and hemisphere, with a large decrease.

### C. Focal MC Model Evokes GC Distortions

We simulated ∂LFP at various impedance mismatches to observe distortions to the PSDs (Figure 5). In addition to the stimulation shaping harmonics (SSH) and the aliased stimulation harmonics (ASH), resulted in a distinct pattern of narrowband peaks when stimulating at 130 Hz and sampling at 422 Hz: largest at 32 Hz, 64 Hz, and 66 Hz. IMH at 66 Hz, specifically, is indicative of MC where the ASH 64 Hz would be present even in the absence of any gain compression. Interestingly, MC induces an absolute reduction of the simulated constant 15 Hz neural oscillation as a function of stimulation voltage (Figure 6a). Other artifacts are evident *in vivo* that are not generated by the MC model (Figure 5f).

### D. In vitro Resistivity Mismatches Distort ∂LFP

To experimentally verify the MC hypothesis, *in vitro* ∂LFP recordings are measured at different impedance mismatches. Measurement in uniform saline are consistent with the predicted ASH and IMH artifacts, particularly the IMH at 66 Hz (Figure 5e,f). Measurement at the interface of saline and agar exhibited larger impedance mismatch and larger artifacts, confirming sensitivity of these distortions to impedance mismatch alone (Figure 6c,d). The distinction between the SAH 64 Hz artifact and the IMH 66 Hz is evident as a broader multipeak artifact, present in both *in silico* (Figure 5e) and *in vitro* models (Figure 5f). Recordings during the voltage sweep confirmed further sensitivity of the distortions to stimulation voltage at a fixed impedance mismatch, and the voltage sensitivity is stronger in the interface recordings than the uniform recordings (Figure 6d). Oscillatory powers calculated in each *in vitro* mismatch condition reflected the level of MC, with interface recordings having broadly higher measured power in all bands (Figure 6e,f). Note the presence of peaks that do not clearly change as a function of stimulation amplitude - such as the 60 Hz noise typical of power grids.

### E. Assessing Gain Compression Ratio (GCr)

**Fig. 2.**
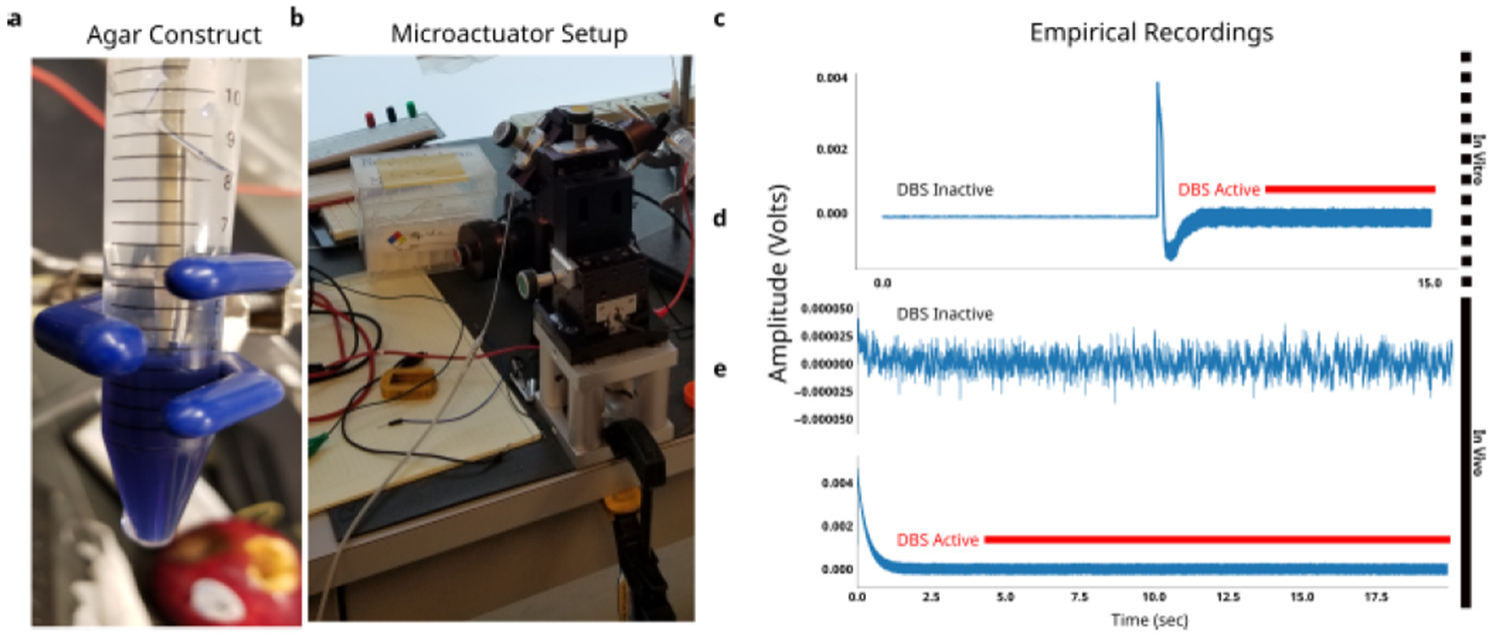
Empirical ∂LFPs. **a**, *in vitro* agar construct with two distinct phases - saline (clear) and agar (blue). DBS lead is placed in saline phase. **b**, DBS lead is fixed at either uniform saline or interface conditions with microactuator rig. **c**, A 15 s *in vivo* recording from Activa PC+S^™^ without active stimulation. **d**, A 15 s *in vivo* recording with active stimulation. Recording settling time was seen in first few seconds. **e**, Stimulation onset during voltage sweep shows stimulation transient.

GCr calculated under each stimulation voltage for both the uniform (low impedance mismatch) and interface (high impedance mismatch) conditions (Figure 6g,h). Using the GCr, we observed a large difference between impedance mismatch conditions within a fixed stimulation voltage (Figure 6i). The magnitude of this difference related to the stimulation amplitude non-linearly (Figure 6i) - 2 V stimulation at the interface condition demonstrated a larger GCr magnitude than 8 V at uniform condition.

### F. Validating Mitigations

#### 1) Uncorrected MC:

Both *in silico* and *in vitro* ∂LFP identifies broad-band and narrow-band distortions that are sensitive to impedance mismatch and stimulation amplitude (Figure 5 and Figure 6). A mitigation pipeline for MC is developed for the case of 130 Hz to avoid features that can be distorted (See Methods II-H).

#### 2) In Vitro Validation:

The MC mitigation pipeline is validated using *in vitro* recordings during the voltage sweep (See Section II-C.3). The complete pipeline applied to *in vitro* voltage sweep normalized oscillatory power across different stimulation conditions (Figure 7a,c). Residual variability in *θ* is evident, but the variability is much smaller than the variability without mitigation and in the reverse direction (Figure 7b vs Figure 7d).

#### 3) In Vivo Application:

We applied the mitigation to *in vivo* clinical recordings to demonstrate the effects on measured PSDs with MC (Figure 7e,f). In both patients, PSDs are brought into alignment through the removal of broad-band slope, and artifacts are avoided by adjusted oscillatory bands (Figure 7g,h).

## IV. Discussion

Chronic measurements from ∂LFP recordings are becoming critical tools in engineering long-term disease readouts for slow-moving brain disorders [22], [36]. In this study, we confirmed that oscillatory power features from ∂LFP recordings can be distorted by *mismatch compression* and propose a novel approach to mitigating these distortions. The resulst enable more reliable analyses, while providing an open-source set of tools for generalizing and extending MC mitigation.

### A. ∂LFP Recordings Consistent With Gain Compression

Oscillations in LFP reflect large-scale synaptic inputs into gray matter and correlate with function [13], [37], suggesting a first approach to disease readouts. We observed large ∂LFP changes in clinical *in vivo* recordings, both acutely with active stimulation and chronically with unclear correlates. Analyses demonstrated frequency-domain changes that were consistent with gain compression, expected by PC+S^™^ engineers [7], but more focused characterization and mitigation was needed to ensure impedance mismatches were not confounded with neural correlates of depression. An expected failure mode of the ∂LFP channel in the Active PC+S^™^ is asymmetries in the recording electrodes leading to imperfect stimulation rejection, and subsequent saturation of recordings [7], [12]. One approach is to only analyze recordings without active stimulation, and our group’s work have identified potential depression readouts here [4], [38]. However, brain dynamics alongside active DBS may contain disease-critical information, like a running car provides more informative signs of underlying problems versus a car turned off.

### B. Impedances Can Be Mismatched and Dynamic

Heterogeneity of brain tissue around the recording electrodes, in particular between gray and white matter, is expected to yield differences in electrical impedance and differences in dynamics of those impedances [4], [16], [17], [23], [39], [40], [41], [42]. Analyses of volume of tissue activated (VTA) has shown clear, unavoidable heterogeneity in the composition of brain tissue around DBS electrodes, strongly suggesting unavoidable impedance mismatches [4], [23]. The SCCwm target has been well studied for antidepressant applications [2], [24], [43] and is a white matter target with gray matter around it [4]. VTA analyses demonstrate variability in the white matter tracts being modulated [23] and future studies may enable more explicit incorporation of VTA analyses to mitigate mismatch through optimizing targeting for higher recording fidelity. In both impedance mismatch and ORM measurements, two distinct phases were consistently seen across patients: a variable phase (0-10 weeks) and a stable phase (11-28 weeks), with significant variability between patients through all phases (Figure 4c,d). The mere presence of impedance mismatches that change over time raises concern for changing levels of artifact rejection; the simultaneous observation in clinical recordings of slope flattening and narrowband artifacts raised strong suspicion for MC distortions. Confirming and mitigating MC distortion is critical to ensuring any derived readout reflects neural oscillations related to depression, and not asymmetric tissue changes occurring at the same timescales of symptom recovery.

### C. Focused MC Model Explains Benchtop Measurements

MC is expected as a failure mode in PC+S^™^ recordings [7] and we developed an equivalent circuit for our focused MC model (Figure 5). The central driver of MC is impedance mismatch between the recording electrodes, and simulated ∂LFP at differing impedance mismatch resulting in frequency-domain changes similar to those seen *in vivo*. Since we used a phenomenological LFP model with a simplified, but still congruent, stimulation waveform, we were able to confirm the presence of MC through the difference between ASH (64 Hz) and IMH (66 Hz) power (Figure 5e). By changing the impedance mismatch through an *in vitro* preparation and observing distortions associated with gain compression, as predicted by the focused MC model (Figure 5f), we confirmed the presence of MC in ∂LFP recordings taken with the Active PC+S^™^. In particular, the emergence of IMH, which trace back specifically to the clipping phenomena linked to gain compression (Figure 1d), supports the presence of MC specifically, over higher-order interactions. While a zero-mismatch condition was unattainable, likely because of differences in the internal wire resistance from electrodes to the IPG, the observation that increases in mismatch yielded the predicted broad- and narrow-band changes of GC was strong evidence of the expected MC. Every artifact predicted in the *in silico* model was present in the *in vitro* model and sensitive to impedance mismatch, confirming the presence of MC. Other peaks were apparent on visual inspection of *in vitro* recordings under all conditions, including 0 V and are considered device-specific artifacts not directly arising from mismatch compression. More sophisticated models can be integrated into the MC simulator codebase to predict distortions at different stimulation frequencies and in different devices, and to explain putative artifacts not explained by MC (Figure 5f; 22 Hz to 25 Hz). However, given the a priori expectation of MC and the alignment of *in silico* and *in vitro* changes, we can confirm that MC is distorting recordings.

### D. Preprocessing Removes Frequency-Domain Distortions

**Fig. 3.**
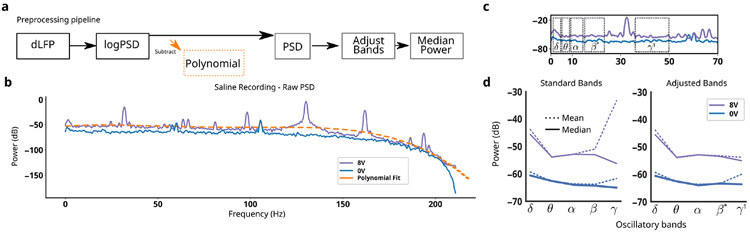
MC Mitigation Steps - **a**, MC mitigation pipeline for ∂LFP recordings remove frequency features that can be distorted. **b**, Empirical PSD in saline at two stimulation voltages demonstrates mismatch compression. **c**, Adjustments to the frequency windows for oscillatory bands to avoid mismatch compression artifacts. In this illustration, no polynomial subtraction is performed. **d**, Comparison of mean and median power calculation within standard and adjusted bands. Calculations are performed in both 0V and 8V stimulation.

**Fig. 4.**
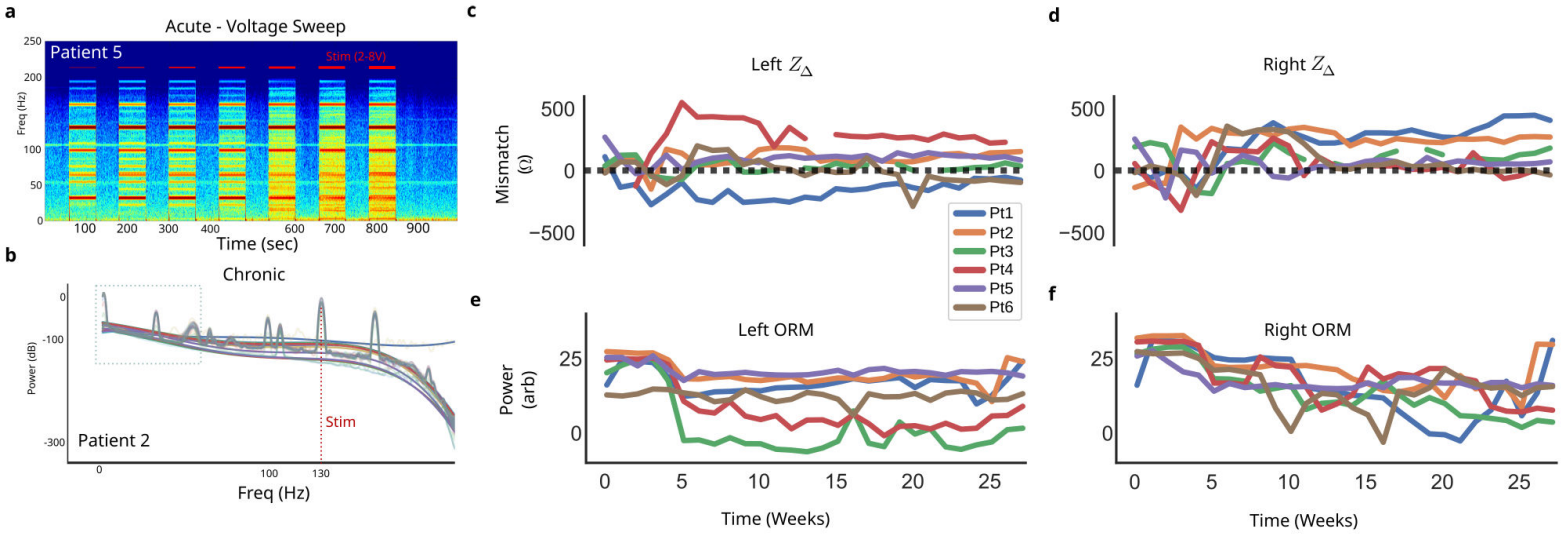
*In vivo* measurement variability. **a**, Spectrogram of *in vivo* voltage sweep from 2V to 8V demonstrates significant changes locked to stimulation. **b**, Weekly averaged ∂LFP PSDs over seven months in a single patient demonstrate significant variability across months of recording. Each bold color curve (translucent) is a fourth-order polynomial fit to the weekly average (thin color curve). **c**, Impedance mismatches in recording electrodes of all patients demonstrate large, dynamic mismatches in left and **(d)** right DBS leads. **e**, An over-range marker (ORM) power was calculated across recordings from all weeks to assess presence of overt gain compression (saturation), demonstrating variability over the weeks in both left and **(f)** right channels.

**Fig. 5.**
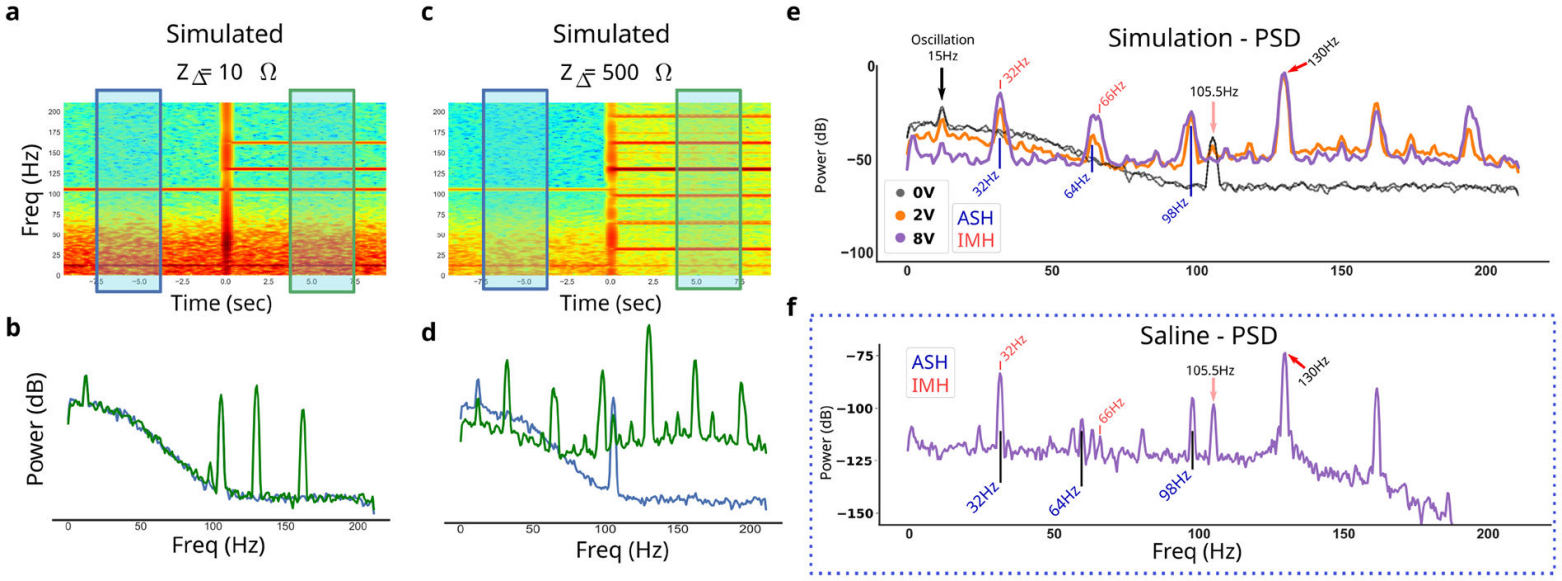
Simulation generates gain compression distortions with impedance mismatch. **a**, Simulated ∂LFP at a low impedance mismatch (*Z*_Δ_) shows artifacts during simulated 130 Hz stimulation. Before stimulation (blue box and line) is compared to during stimulation (green box and line). **b**, Power spectral density (PSD) for the before and during stimulation time periods. **c,d**, Simulated ∂LFP at a high impedance. **e**, Simulated PSD at various stimulation voltages. Aliased simulation harmonics (ASH) and intermodulation harmonics (IMH) are labeled. **f**, Empirical recording in saline shows an observed peak at all simulated peaks. Additional peaks are present and not attributed to MC due to their insensitivity to impedance mismatches.

**Fig. 6.**
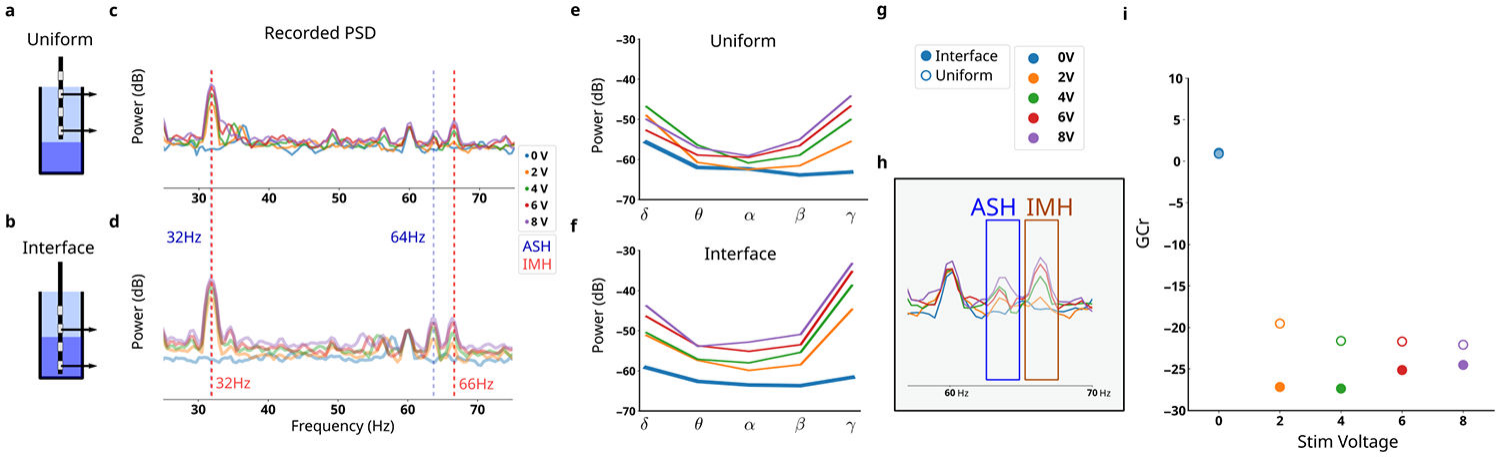
*In vitro* Mismatch Compression. ∂LFP recordings were captured in two configurations: **a**, uniform saline medium and **b**, interface of saline-agar. **c**, Uniform-medium PSDs at various voltages demonstrate distinct peaks, with 32 Hz, 64 Hz, and 66 Hz being highlighted for their voltage-dependence. **d**, Interface-media PSDs demonstrate more voltage-dependence. **e**, Oscillatory power calculated in uniform-medium at various stimulation voltages compared to **f**, interface-medium. **g**, Recordings taken at both interface (solid dot) and uniform (empty dot), with a range of stimulation voltages 0V to 8V. **h**, Aliased stimulation harmonic (ASH) arise from suboptimally sampled stimulation shaping harmonics (SSH), while intermodulation hamornics (IMH) arise directly from amplifier gain compression. **i**, Gain compression ratio (GCr) calculated from ASH and IMH reflect both stimulation voltage and impedance mismatch.

**Fig. 7.**
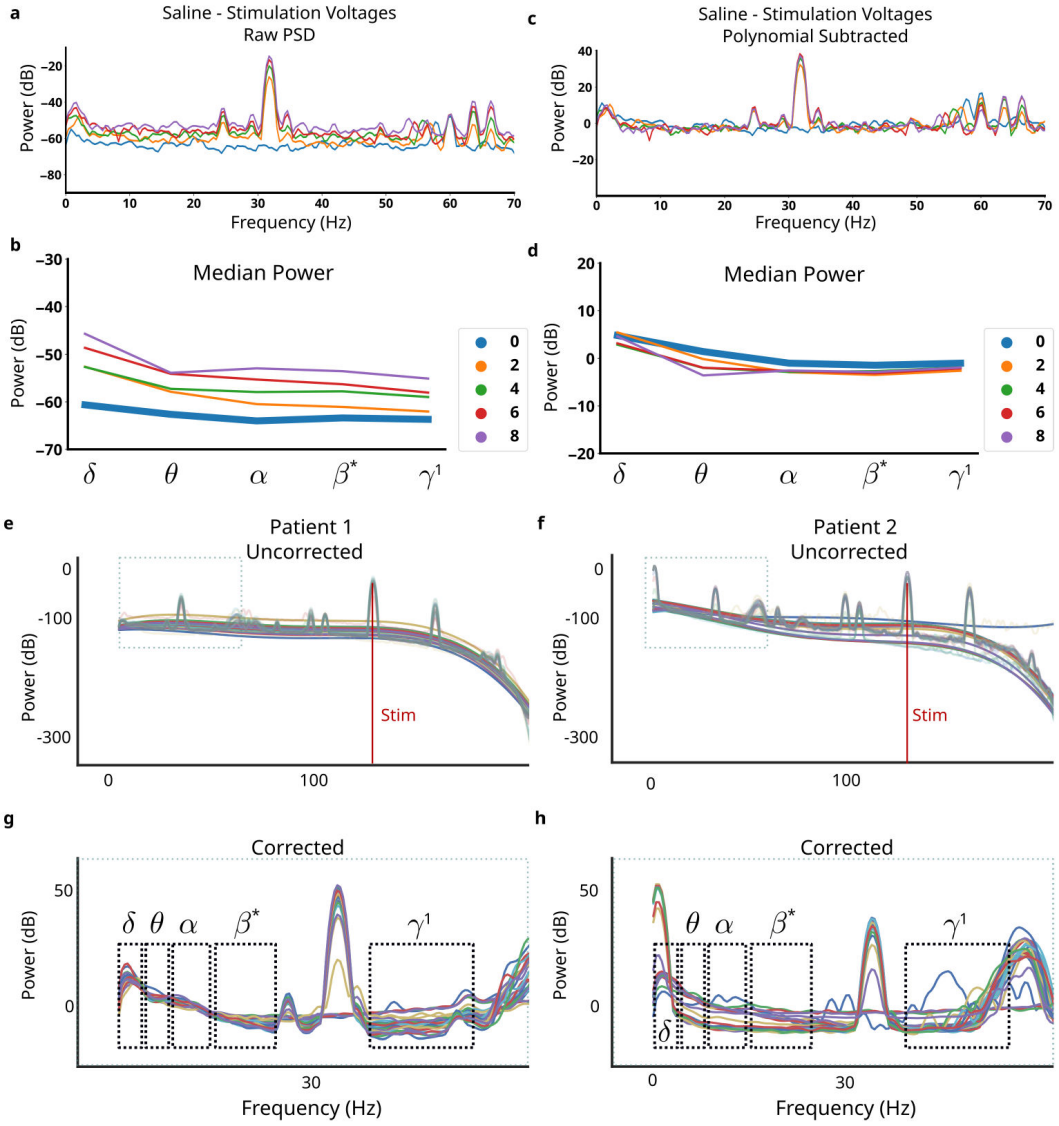
Applied MC Mitigations. **a**, Raw PSDs recorded in agar at stimulation voltages between 0V to 8V. **b**, Oscillatory power calculated in each band for each tested stimulation voltage. **c**, Corrected PSDs remove features that are corrupted by mismatch compression. **d**, Oscillatory power calculated converges to the no-stimulation condition across all stimulation voltages. **e,f**, PSDs from chronic recordings across 7 months in two patients. Thin lines are weekly averaged PSDs, bold lines are polynomial fits to highlight weekly variability. **g,h**, MC pipeline applied to all weekly PSDs shows removal of features distorted by MC. Significant inter-patient variability is observed; differences in oscillatory power can more confidently be ascribed to neural sources.

Minimizing the effect of MC distortion is crucial for a reliable long-term disease readout in ∂LFP recordings, but inverting it after data collection is impossible in the presence of noise. Our *in silico* model identified MC relevant distortions that were used to remove broad-band distortions and adjust the traditionally defined oscillatory bands (Table III). Removal of the broadband-slope yielded large improvements in the observed MC distortion (Figure 7b vs Figure 7d). Band ranges were chosen using both *in silico* and *in vitro* LFP during stimulation to find the maximal continuous range within standard oscillatory bands that also avoided ASH+IMH (Figure 6 vs Figure 4b). The adjusted bands remain inside their associated windows, enabling more congruent interpretation in the context of the broader literature. β*, for example, is a subset of *β* that is aligned with definitions of low-*β* found in Parkinson’s Disease DBS [44], [45]. β* was further constrained due to a PC+S^™^ artifact that was not linked to MC, and further investigation is needed to identify its source, but this step continues ignoring MC-related artifacts. This mitigation pipeline yielded oscillatory power calculations that converged across all stimulation voltages, leading to more accurate measurement (Figure 3). We then observe its effects on *in vivo* recordings from two patients, seeing PSDs across 24 weeks converge despite known impedance mismatches (Figure 7g,h).

### E. Limitations

First, the reduced ∂LFP model does not recapitulate all device-specific artifacts and is, therefore, only one part of mitigating all potential distortions [9], [46]. Addition-ally, our phenomenological generation of ∂LFP limits the ability to link post-MC mitigated observations to neural generators in favor of focused characterization of MC. Second, the tissue-electrode interface ignores capacitance in impedance [47] and does not account for distortions in phase spectrum, only power spectral analyses. Third, the mitigation pipeline avoids features susceptible to MC distortion, potentially removing important neural activity in the $\frac{1}{f}$ slope feature and in important frequency sub-windows [4], [31], [45]. Our proposed restriction on oscillatory band ranges limits subsequent generalization to the broader ranges established in the literature. Finally, we study and model MC in the presence of DBS artifact but other large-amplitude artifacts can drive MC, such as ECG and EMG signals evident in the PC+S^™^ [9]. Any large-amplitude artifact measured the tissue around the DBS lead can drive MC, with oscillatory distortions specific to the spectral content of the artifact.

### F. Impact

The growth of *connectomics* guided DBS, especially in psychiatric indications, highlights the importance of accounting for tissue heterogeneity in ∂LFP recordings from gray matter when deliberately targeting white matter [14], [15], [20], [23], [41]. Given the variability in anatomy between hemispheres, patients, and targets, it’s unlikely that this mismatch can ever be avoided completely, and this work provides a needed first-characterization of a distortion process intrinsic to all ∂LFP recordings. While next-generation devices have gone a long way to improving recording hardware [8], [10], [46], MC remains a concern in any ∂LFP device. Care must be taken to rule out MC in any readout applications, acute or chronic, if using a ∂LFP recording channel [10], [46]. Hardware-based improvements are certainly needed, and techniques like artifact blanking may help avoid amplifier distortions at the cost of missing key time windows for DBS effects [45], [48]. Even with hardware improvements, analytical mitigation strategies may enable low-cost bdDBS devices that can achieve similar readout utility. Ultimately, this work enables more reliable oscillatory analyses using ∂LFP devices, including first-generation Active PC+S^™^, particularly using supervised machine learning that leverages behavioral signals to isolate neural readouts [49].

## V. Conclusion

Impedances mismatches in ∂LFP recordings can cause imperfect rejection of therapeutic stimulation and result in gain compression distortions, a process we characterize here as mismatch compression (MC). MC distorts specific frequency-domain features of recordings, specifically broad-spectrum $\frac{1}{f}$ slope and narrowband artifacts at predictable frequencies. These distortions can be mitigated by ignoring features that are susceptible to distortion, a cautious approach that enables analysis of suboptimal recordings from a growing class of white-matter targets in psychiatric disorders, such as the SCCwm in depression studied here. Our results yield tools to improve development of reliable disease readouts, especially during active stimulation, that are critically needed in growing use of ∂LFP recordings. Future work focused on explicit inversion of MC distortions through models, like the one developed here, can enable low-cost adaptive DBS platforms that remain robust in slow-moving disease recovery and chronic timescales.

## Figures and Tables

**TABLE I:** Patient Demographics and Parameters. Patient Demographics and Recording Channel Parameters

Patient	Age	Sex	Recording Electrodes	Gains
Patient 1	50	F	(E1,E3)+(E8,E10)	250,2000
Patient 2	48	F	(E1,E3)+(E9,E11)	1000,1000
Patient 3	70	F	(E1,E3)+(E8,E10)	2000,2000
Patient 4	64	M	(E1,E3)+(E9,E11)	2000,2000
Patient 5	62	F	(E0,E2)+(E8,E10)	250,250
Patient 6	57	M	(E1,E3)+(E8,E10)	250,250

**TABLE II:** Model Parameters Used for Study Simulations

*A*_*d*_	*Z*_*b*_	$\frac{1}{f}\;\text{Strength}$	*x*_1_ amplitude	*g*1	*g*2
0.5	1 · 10^4^Ω	1 · 10^−3^	2 · 10^−3^	1	1

**TABLE III:** Summary of Oscillatory Band Adjustments for MC Mitigation. Shifted Oscillatory Band Frequency Windows Mitigate MC Distortions While Retaining Interpretability

	δ	θ	α	β	γ
Standard	1 Hz to 4 Hz	4 Hz to 8 Hz	8 Hz to 14 Hz	14 Hz **to** 30 Hz	30 Hz **to** 50 Hz
Adjusted	1 Hz to 4 Hz	4 Hz to 8 Hz	8 Hz to 14 Hz	14 Hz to 20 Hz	40 Hz to 50 Hz
